# TS-1/spherical activated carbon composites in the epoxidation of methyl oleate†

**DOI:** 10.1039/d4ra08189g

**Published:** 2025-03-05

**Authors:** Adrián Osorio Hernández, Michael Goepel, David Poppitz, Muslim Dvoyashkin, Roger Gläser

**Affiliations:** a Institute of Chemical Technology, Universität Leipzig Linnéstr. 3 04103 Leipzig Germany michael.goepel@uni-leipzig.de

## Abstract

One approach to increase the catalytic efficiency of TS-1 is the synthesis of TS-1 composite materials together with spherical activated carbon (SAC). Although increased Ti-site normalized catalytic activity of such composites compared to parent materials was observed, the reason for the increased activity is not fully understood. This study therefore aims to correlate the physico-chemical and catalytic activity of TS-1/SAC composites. Composite materials with different TS-1 weight fractions were prepared and applied in the heterogeneously catalyzed epoxidation of methyl oleate using aqueous H_2_O_2_. Up to seven times greater Ti-site normalized activity for the composites was observed compared to the parent TS-1. The pulsed field gradient (PFG) NMR was then used to rationalize the catalytic activity results. SAC was found to function similarly to a conventional catalyst support (*e.g.*, in supported metal catalysts) for the TS-1. The SAC thus allows control of the dispersion of and pore space arrangement between TS-1 crystals, which could be directly linked to an increase in catalytic activity. In addition to gaining insight into the fundamental principles of such composite catalysts, the importance of the sorption of reactant and product molecules on the catalytic activity was pointed out.

## Abstract 2

Different diffusion regimes observed in methyl oleate epoxidation are correlated with catalytic activity. The complex composite catalyst can be understood as TS-1 crystallites as “active sites” on a spherical activated carbon (SAC) support.

## Graphical abstract

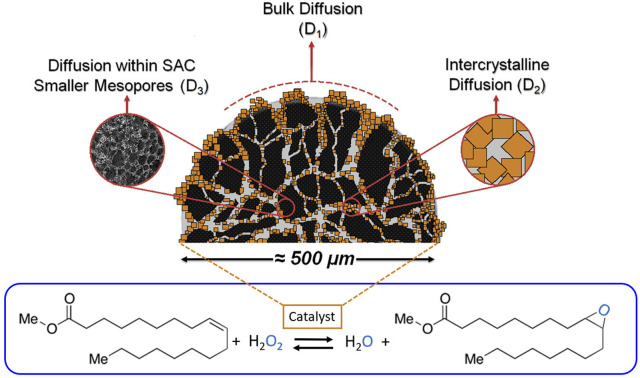

## Introduction

Titanium-silicalite-1 (TS-1) with MFI-type framework topology was synthesized for the first time in 1983 by Taramasso *et al.*^1^ Since then, the catalytic properties of Ti-containing silicalites have been extensively investigated, particularly because of their high selectivity for heterogeneously catalyzed green oxidation reactions using hydrogen peroxide (H_2_O_2_) as an environment-friendly oxidizing agent.^2^ Due to its established catalytic properties, TS-1 has been used as an efficient and selective catalyst in the epoxidation of alkenes,^3,4^ in particular, propene,^5,6^ hydroxylation of aromatic compounds,^7^ ammoxidation of cyclohexane,^8^ oxidative desulfurization,^3,9^ and oxidation of alkenes and alcohols. TS-1 is synthesized hydrothermally, in which part of Si^4+^ is isomorphically substituted by Ti^4+^ during preparation.^2^ Almost all Ti species are present in the Ti^4+^ state, although a small portion may also be present as Ti^6+^, Ti^5+^, and TiO_2_ (anatase).^10^ However, there are some drawbacks to the use of TS-1 as a catalyst. On the one hand, the incorporation of titanium in the MFI framework is limited to a maximum Si : Ti atomic ratio of 100 : 3 since above this range titanium segregates as TiO_2_.^11^ On the other hand, the width of its micropores limits the accessibility of species with a molecular diameter larger than 0.55 nm.^12^ To solve this issue, many methodologies have been developed to create a secondary pore system with a larger diameter within the crystals. These methodologies are divided into two main approaches: top-down and bottom-up.^13–15^ In the former, the secondary pore system is introduced by subjecting the microporous material to post-synthetic modifications, such as alkali treatment, acid treatment, and surfactant templating.^16–21^ In the latter approach, a secondary pore system is introduced during the initial synthesis of the catalyst using different structure-directing agents (SDA).^22–27^ In both cases, these procedures lead to the generation of a secondary pore system in meso- and macropore order.

In addition to pore system modifications, several studies have focused on modifying other properties of TS-1, such as surface hydrophobicity to improve its catalytic activity during reactions involving hydrophobic organic molecules.^28^ For example, With *et al.* combined TS-1 with a spherical activated carbon (SAC) because of its hydrophobic surface, high surface area, and micro-, meso- and macropores can facilitate the adsorption of organic molecules like fatty acid methyl esters (FAMEs).^29^ In most studies reported in the literature on the epoxidation of FAMEs, methyl oleate (MO) is commonly used as a model compound for fatty acid methyl ester because of its representative chemical features and facile availability.^30,31^ Also, the dimensions of the MO are representative of biomass-based molecules and are relevant in the framework of mass-transfer limitations in nanoporous catalysts. The composite catalyst made of TS-1 and SAC showed up to four times more activity (normalized to number of Ti sites) than a commercial TS-1 during epoxidation of methyl oleate with H_2_O_2_ to epoxidized methyl oleate (eMO). The increase in activity was attributed to the adsorptive enrichment of the reactants in the vicinity of the active Ti sites owing to the presence of SAC. However, the precise role of SAC in the TS-1-catalyzed liquid-phase epoxidation is inconclusive, as any increase in catalytic activity may also be attributed to the increased accessibility of Ti active sites.^32^ In this context, pulsed field gradient nuclear magnetic resonance (PFG NMR) is a useful method for probing the diffusion properties of solid catalysts, particularly in the presence of confined geometries.^33^*In situ* diffusion studies are rare for FAMEs due to their large molecular size, which limits research into their interaction with active sites. However, the demand for FAME-derived epoxides highlights the need for catalytic testing. Therefore, this study uses the epoxidation of methyl oleate, a bulky molecule commonly used to assess the catalytic behavior of such composites. Previously, Dvoyashkin *et al.*^34^ studied the diffusion of MO in oversaturated pelletized micro- and mesoporous TS-1 using PFG NMR. Three diffusion regimes were identified: (i) bulk-like and/or film diffusion outside the pellets, (ii) diffusion between TS-1 crystals within individual pellets, and (iii) diffusion inside mesopores of the TS-1 crystals. The intracrystalline diffusivities were approximately two orders of magnitude lower compared to those of diffusion in the bulk-like phase at the same temperature. In the parent microporous TS-1, no diffusion of MO within the crystals was observed due to limited accessibility.^34^

Based on the improved catalytic activity of TS-1/SAC composites observed by With *et al.*,^29^ in this work, the synthesis of a range of such composites is combined with catalytic studies for MO epoxidation to eMO, along with PFG NMR studies. Therefore, the aim was to rationalize the observed increased activity (Ti-site-normalized) in terms of the composite catalyst structure and morphology and the influence of the modified pore characteristics of the parent TS-1 material on its diffusion properties. For this, composites of TS-1 and SAC with different TS-1 weight fractions were prepared and characterized extensively to determine the spatial arrangement of the TS-1 in the composite materials. Then, PFG NMR was used to assign observed diffusion regimes to a certain catalytic activity of a material. Furthermore, the role of sorption of reactants with respect to the increased catalytic activity, as proposed by With *et al.*, was investigated in more detail.

## Experimental section

### Synthesis of TS-1/SAC composites

**Fig. 1:**
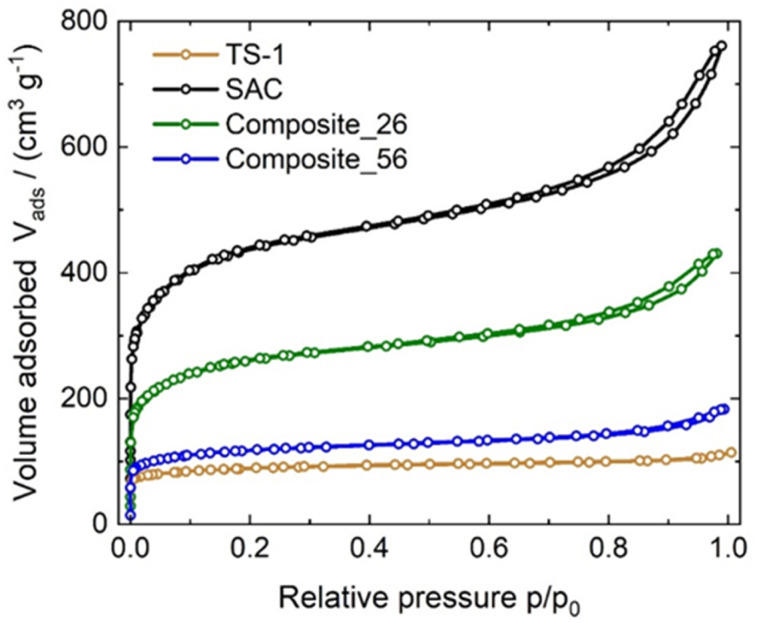
Nitrogen sorption isotherms displayed as volume adsorbed (*V*_ads_) over relative pressure (*p*/*p*_0_) of TS-1, SAC, Composite_26 and Composite_56 recorded at 77 K.

The syntheses of TS-1 and the two composite catalysts of TS-1 and SAC (Composite_26 and Composite_56) were carried out according to the procedure reported by With *et al.*^29^ Numbers 26 and 56 represent the weight fraction of TS-1 (wt%) in the synthesized composites. The weight fraction of 56 wt% TS-1 was the maximum that could be synthesized on the SAC surface and pores; TS-1 is deposited outside the SAC structure from this weight fraction. Briefly, the hydrothermal synthesis of TS-1 was carried out in a PTFE-lined stainless-steel autoclave (*V* = 45 cm^3^) in the presence of either 5.1 g or 1.4 g of SAC (*S*_BET_ = 1564 m^2^ g^−1^, *V*_total_ = 1.80 cm^3^ g^−1^, *d*_particle_ = 450–500 μm, provided by Blücher GmbH) at 438 K for 96 h. The molar composition of the resulting synthesis gel was 1 TEOS : 0.014 TIP : 0.2 TPAOH : 22.2 H_2_O, where TEOS is tetraethylorthosilicate, TIP is titanium(iv) isopropoxide, and TPAOH is tetrapropylammonium hydroxide. The reaction mixture was quenched by cooling the autoclave to <313 K in water within 10 min. The resulting solid was separated from the reaction mixture by centrifugation, washed twice with 30 cm^3^ deionized water, dried for 24 h at 393 K in air, and finally calcined at 573 K in air-flow (150 cm^3^ min^−1^) to obtain the TS-1/SAC composite catalysts. It has been previously shown that SAC is thermally stable at the applied calcination temperature.^35^ This was also confirmed in the present study by thermogravimetric analysis (TGA) (Fig. 1S†).

### Catalyst characterization

The TS-1 and TS-1/SAC composites were characterized by nitrogen sorption, elemental analysis *via* optical emission spectrometry with inductively coupled plasma (ICP-OES), mercury intrusion porosimetry (MIP), scanning electron microscopy (SEM), energy dispersive X-ray (EDX) analysis, powder X-ray diffraction (XRD), thermogravimetric analysis (TGA), and diffuse reflectance UV-Vis spectroscopy (DR-UV-Vis).

BELSORP®-miniX (Microtrac Retsch GmbH, Haan, Germany) was used to record the nitrogen sorption isotherms. The samples were evacuated at 523 K under a vacuum of 3 × 10^−11^ MPa for 24 h before the measurements. The isotherms were measured at 77 K. The total specific surface area (*S*_BET_) was determined using the Brunauer–Emmett–Teller (BET) model from the adsorption branch of the isotherm in the relative pressure range of *p*/*p*_0_ = 0.01–0.10. The total specific pore volume (*V*_total_) was estimated from the total nitrogen uptake at a relative pressure (*p*/*p*_0_) of 0.99.

Ti-content was determined by ICP-OES using an Optima 8000 spectrometer (PerkinElmer, Waltham, USA). Before the analysis, the samples were dissolved in 2.0 cm^3^ HF, 3.0 cm^3^ HNO_3_, and 3.0 cm^3^ HCl and diluted to obtain an aqueous solution of 20 cm^3^ which also contained 12.0 cm^3^ H_3_PO_4_ for complexation of excessive HF.

MIP was performed using a PoreMaster 60 (Quantachrome Instruments, Boynton Beach, FL, USA) from 0.02 to 400 MPa. The mercury contact angle was set at 141°, and a surface tension of 0.48 N m^−1^ was used to determine pore widths, porosities, and specific pore volumes. Pore width distribution in MIP was determined using the Washburn equation, assuming cylindrical pores in the software package SOLID-SOLver of Intrusion Data (Version 1.6.6).

SEM was performed using a Leo 1530 device (Zeiss, Oberkochen, Germany). The samples were placed on carbon tabs and glued with carbon paste. For conductivity, the samples were sputtered with gold or carbon and investigated under a high tension of 5 kV. The particle widths were calculated by taking the average of 100 optically determined particle widths from the SEM images of each sample. Energy-dispersive X-ray spectroscopy (EDX) was performed using an Oxford Instruments device (model no. 7426) with an energy resolution of 138 eV. EDX was used to determine the carbon (C), silicon (Si), titanium (Ti), and oxygen (O) contents in atomic percent (atom%).

The powder XRD patterns were recorded at room temperature using a G670 diffractometer (Huber, Rimsting, Germany). The diffracted intensity of Cu-Kα radiation (*λ* = 0.154 nm) was measured in the range of 2*θ* between 4° and 90°, with a step size of 0.005° and a counting time of 0.2 s for phase identification.

The SAC and TS-1 weight contents of each composite were obtained using differential thermal analysis (Netzsch STA 409 TG/DTA, Stuttgart, Germany) by differential weighing after burning the SAC from room temperature to 873 K in air with a ramp rate of 10 K min^−1^.

Diffuse-reflectance UV-Vis spectroscopy was used to determine the Ti species present in the materials. The experiments were performed using a Lambda 650S UV-Vis spectrophotometer (PerkinElmer, Waltham, USA). Spectra were recorded in the range of 190 to 800 nm with a step width of 1 nm and a slit width of 2 nm. A Spectralon disc (Labsphere) was used as reference material.

### PFG NMR experiments

Diffusion experiments were carried out by ^1^H NMR using a wide-bore 100 MHz home-built spectrometer at 323 K, as described in.^34^ Approximately 200 mg of each material was placed into an NMR tube and evacuated overnight under 7 mbar and 373 K. Each sample was then loaded with methyl oleate (MO, 99% Sigma Aldrich) until it completely covered the material in the NMR tube. After such oversaturation, the excess MO was partly removed by the tissue and the tubes were flame-sealed. The obtained NMR signal in the diffusion experiment (*ψ*) was analyzed using the least squares fitting of the sum of exponents with respective pre-exponential factors (*p*_*i*_):1
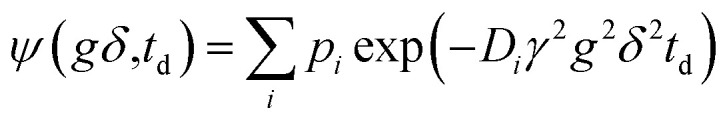
where *D*_*i*_ is the self-diffusion coefficient, *γ* is the gyromagnetic ratio of protons, *g* and *δ* are the amplitude and the duration of the gradient pulses, respectively, and *t*_d_ is the effective diffusion time. The root mean square displacements (RMSDs) were calculated using the Einstein diffusion equation.

### Catalytic experiments

Catalytic experiments were conducted in the liquid phase using a two-neck round-bottom glass flask (*V* = 25 cm^3^) connected to a reflux condenser at 323 K and magnetic stirring (stirring speed of 600 min^−1^) at ambient pressure. In a typical experiment, 10 cm^3^ of acetonitrile (99.9%, VWR) was mixed with 90 mg of MO (≥99%, Sigma-Aldrich) and 150 mg of the catalyst, and the mixture was stirred for 5 h at 323 K before starting the reaction. For this preliminary adsorption experiment, samples of the reaction mixture were taken at 0, 0.5, 1.5, 3, and 5 h. After 5 h, 140 mg of H_2_O_2_ (30 wt% aqueous solution, Sigma-Aldrich) was added to start the reaction, and samples were collected after 5.5, 6.5, 8, and 10 h. The catalyst was removed from the samples by centrifugation. A defined amount of the liquid sample (0.1 cm^3^) was diluted in 0.5 cm^3^ of acetonitrile and analyzed by gas chromatography (Shimadzu GC 2010 equipped with a flame ionization detector). The turnover number (TON) of MO conversion was calculated according to Wilde *et al.*^36^ as moles of epoxy methyl oleate (*n*_eMO_) formed per mole of Ti (*n*_Ti_) present in the catalyst at a given time *t* (TON = *n*_eMO,*t*_*n*_Ti_^−1^).

## Results and discussion

### Textural properties of TS-1 and TS-1/SAC composite catalysts

The textural properties of the synthesized catalysts are listed in Table 1 and the corresponding isotherms are shown in Fig. 1. TS-1 and Composite_56 were identified as type I reversible isotherms, characteristic of microporous solids with relatively small external surfaces. This can be attributed to the fact that microporous TS-1 governs the observed textural properties of the composite, owing to its mass-fraction of 56 wt%. In this context, the observation of a type I isotherm for Composite_56 provides the first indication of possible filling of the pore system of SAC with TS-1 crystallites, as discussed later. Composite_26 and SAC exhibited type IV adsorption isotherms (according to IUPAC classification).^37^ SAC and Composite_26 show an increased uptake at high relative pressures *p*/*p*_0_ > 0.9 accompanied by a type III hysteresis (H3) attributed to the macropores of SAC. The parent SAC showed a specific BET surface area of 1564 m^2^ g^−1^, whereas Composite_26 and Composite_56 exhibited 849 and 412 m^2^ g^−1^ respectively. As expected, the specific surface area of SAC decreased upon the introduction of TS-1, *i.e.*, the higher the TS-1 content, the lower the specific surface area of the composite.

**Table 1:** Specific surface area (*S*_BET_) and specific pore volume (*V*_total_), Ti-content, TS-1 content, and SAC content, for TS-1, SAC and TS-1/SAC composite catalysts

Sample	*S*_BET_/(m^2^ g^−1^)	*V*_total_/(cm^3^ g^−1^)	Ti content/wt%	TS-1 content/wt%	SAC content/wt%
TS-1	210	0.16	2.51	100	—
SAC	1564	1.80	—	—	100
Composite_26	849	1.02	0.51	26	74
Composite_56	412	0.53	1.06	56	44

**Fig. 2:**
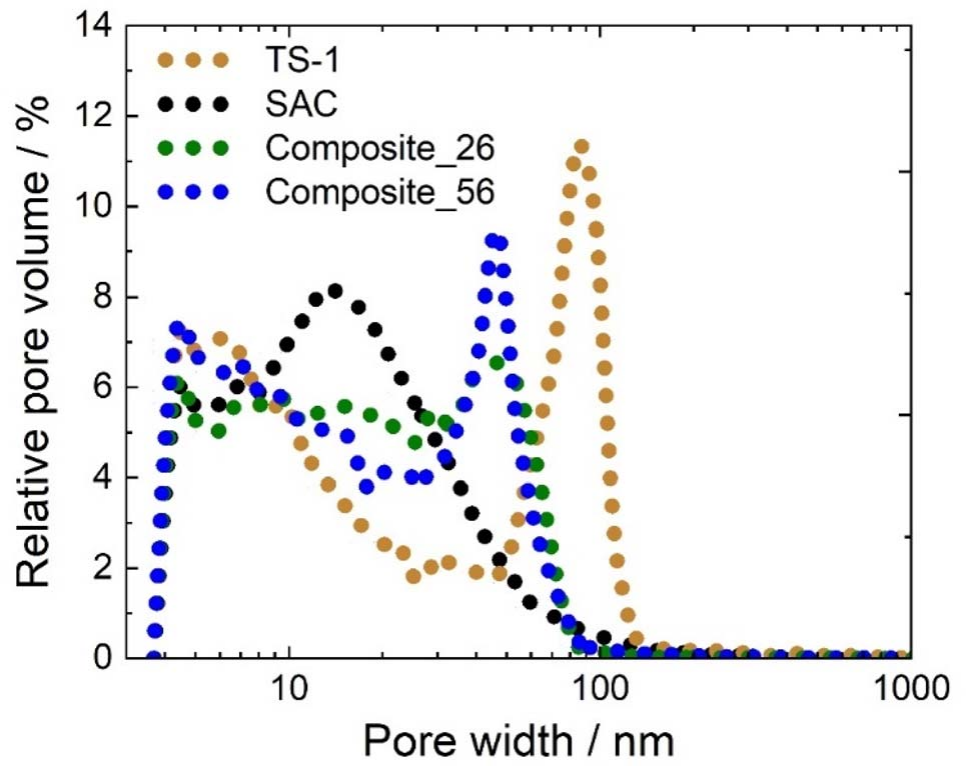
Pore width distribution obtained from Hg-intrusion displayed as relative pore volume over pore width of TS-1, SAC, Composite_26, and Composite_56.

Pore width distributions were further analyzed by MIP. Both composites exhibited mesopores with pore widths between 40 and 50 nm, which were not observed in SAC. These mesopores were probably generated by the stacking of TS-1 crystals, as shown by Malmir *et al.*^38^ in the simulation of porous systems with cubic particles. The MIP results for TS-1 show pores of approximately 100 nm, which are likely the result of the same stacking phenomena of TS-1 crystals but with a larger width because of the lack of confinement within or interaction with the SAC. A reduction in the relative pore volume within the width range of 10–30 nm was observed upon composite formation (Fig. 2). This can be understood as a partial filling of these pores in the SAC when the TS-1 is introduced during composite formation.

**Fig. 3:**
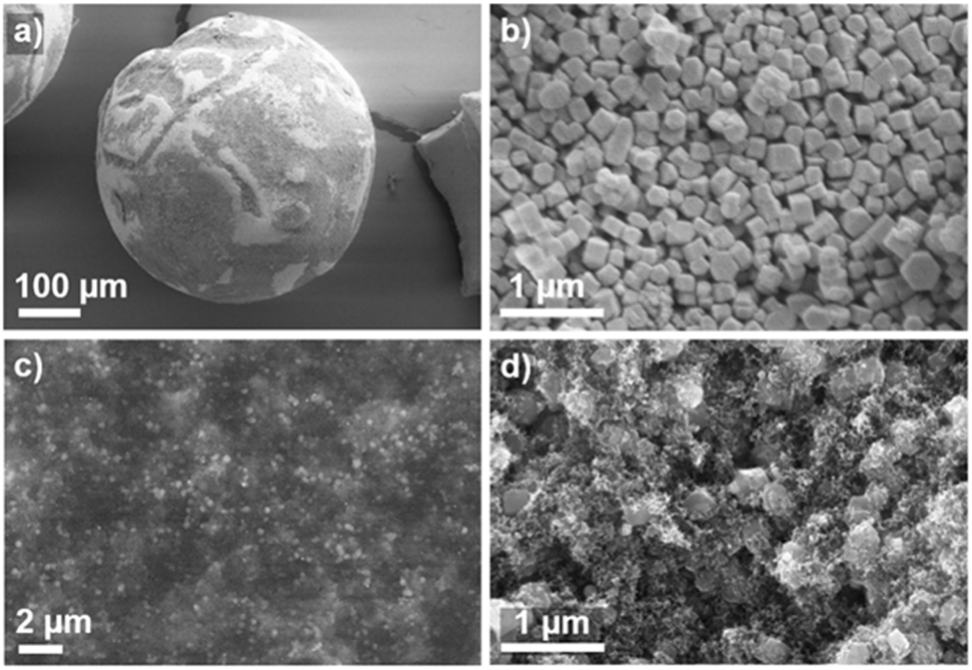
SEM images showing the exterior of a single TS-1/SAC Composite_26 particle (a) and a close-up view of TS-1 crystallites occupying the surface (b). BSE-SEM (c) and SEM detail image (d) of a cross-section of a split spherical particle showing the presence of TS-1 crystallites.

Thus, consistent with the nitrogen sorption results, the MIP results suggest the existence of TS-1 crystals inside the SAC pore system. It should be furthermore noted that the specific pore volumes observed for the composites are lower than what would result from a mass-weighted linear combination of the individual pore volumes of TS-1 and SAC with corresponding weight fractions (Composite_26 : 1.37 cm^3^ g^−1^*vs.* 1.02 cm^3^ g^−1^, Composite_56 : 0.88 cm^3^ g^−1^*vs.* 0.53 cm^3^ g^−1^, see also ESI eqn (1S)†). This again indicates that stacked TS-1 crystals are partially filling the pore system of the SAC, *i.e.* it does not crystallize only on the external surface of the SAC but also inside the pore space of the SAC, which is consistent with observations by SEM (Fig. 3) and XRD data (see Fig. 2S and 3S of ESI†).

**Fig. 4:**
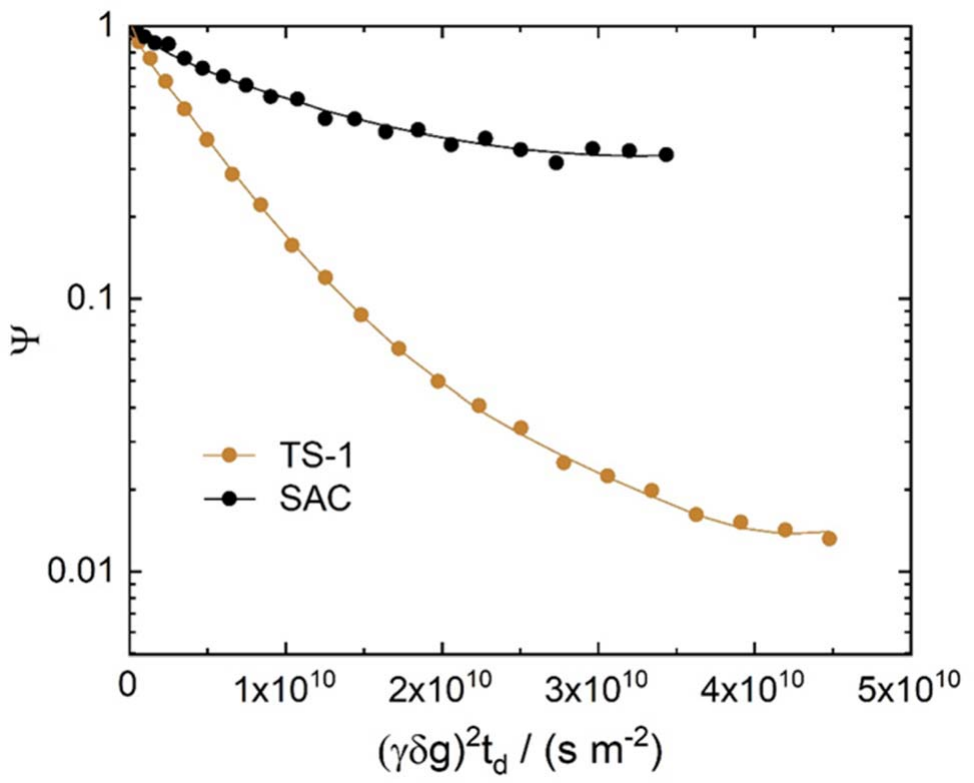
^1^H PFG NMR diffusion attenuation curves obtained for MO in the presence of TS-1 and SAC at 323 K. The solid lines represent the fits using eqn (1) of the data.

**Fig. 5:**
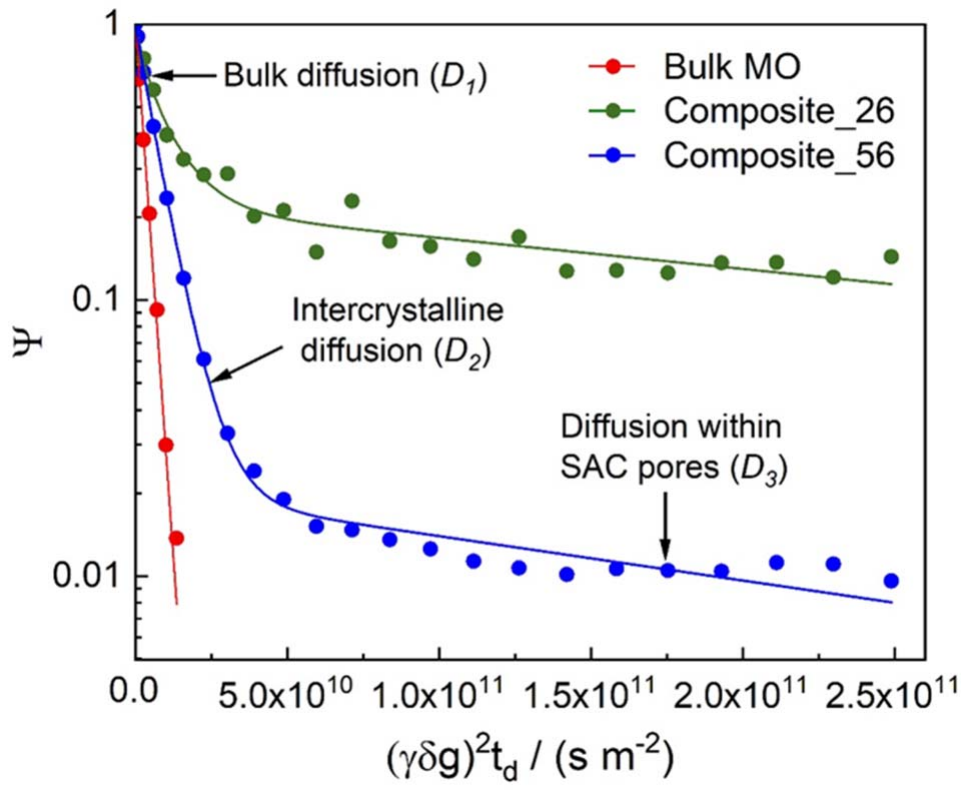
^1^H PFG NMR diffusion attenuation curves obtained for MO in the presence of TS-1/SAC composite catalysts at 323 K. The solid lines represent exponential fits of the data. The arrows point to the different sections of the attenuation curves, in which different diffusion processes dominate: (*D*_1_) – bulk-like and/or film diffusion outside pores, (*D*_2_) – long-range diffusion caused by exchange between the spaces inside and outside pores and/or diffusion in the inter-crystalline space, and (*D*_3_) – diffusion within SAC pores.

Fig. 3 shows SEM images of the synthesized composite catalysts. The obtained composite catalyst particles (Composite_26 and Composite_56) had a diameter distribution of approximately 450–500 μm, showing that the original particle width of the SAC remained unchanged during composite formation (Fig. 4S†). Fig. 3b shows the surface details of a Composite_26 particle, where TS-1 crystals with a mean width of 200 nm can be observed (Fig. 4Sa†). TS-1 crystallization was found to occur both on the outer surface of the SAC and within the pores, as shown by the backscattering electron contrast (BSE-SEM) in Fig. 3c, revealing TS-1 crystals under the surface of the SAC. TS-1 crystals were also identified inside a Composite_26 particle that was split into two, confirming that crystallization occurred on the surface and inside the bulk of the SAC (Fig. 3d). Fig. 5S† shows the surface of Composite_56, showing the TS-1 crystals on its surface, the morphology of a SAC particle and the morphology of the synthesized TS-1 crystals. The composition of TS-1 crystals on SAC was determined by SEM-EDX analysis, and the presence of titanium (Ti), silicon (Si), and oxygen (O) was confirmed (see Fig. 6S of ESI†).

XRD patterns of both composites and TS-1 show characteristic reflections at 2*θ* = 7.9°, 8.9°, 23.1°, 23.9°, and 24.4°, corresponding to the XRD pattern of the MFI-type framework topology with no indication of impurities (Fig. 2S†).^39^ Diffraction patterns of the composites were obtained with and without crushing to further confirm the position of the TS-1 crystals within the SAC. The intensity of the reflections was higher in the crushed samples, indicating that TS-1 crystals were not only found on the surface of the SAC, but also in the interior pore system (Fig. 3S†). The latter is consistent with the observations made using nitrogen sorption, MIP, and SEM-EDX. Further, possible crystal phases, such as extra-framework TiO_*x*_, were below the detection limits and were not detected in these materials by XRD.

The SAC content of the composites was determined by TGA, in which the composites were calcined to 873 K in air at a ramp rate of 10 K min^−1^. The weight losses of Composite_26 and Composite_56 were 74 and 44 wt% respectively, compared to their initial weights. The remaining portion of the sample after calcination was assumed to be TS-1 due to its high thermal stability (Fig. 1S†).

Inductively coupled plasma optical emission spectroscopy (ICP-OES) analysis revealed a Ti-content of 2.5 wt% in the TS-1, 0.51 wt% in Composite_26, and 1.05 wt% for Composite_56. The Ti-content in the composites is directly related to the TS-1 content (Table 1), and the amount of Ti incorporated in TS-1 was not significantly changed upon composite formation. In Composite_26, the Ti-content in the TS-1 is 2.0 wt%, and in Composite_56 is 1.9 wt%. The UV-Vis spectra of the calcined sample (see Fig. 7S†) confirm the incorporation of Ti species in the MFI framework (205-210 nm, corresponding to Ti(OSi)_4_ species), titanol species (Ti(OH) (OSi)_2_ at 228 nm), and a small peak at 320 nm corresponding to TiO_2_.^40,41^ Overall, the UV-Vis data confirm the relatively similar Ti sites in all catalysts.

In summary, the synthesized TS-1 and TS-1/SAC composite catalysts showed an MFI-type framework topology characteristic of the parent TS-1 with high crystallinity, as evidenced by the XRD data. The specific BET surface area and total (accessible) pore volume of the composite catalysts decreased with increasing TS-1 weight fraction. Titanium was present in the composites and incorporated into the MFI framework of TS-1. The TS-1 crystals are located on the surface of the SAC and within the SAC pores, as evidenced by textural analysis, SEM images, BSE-SEM, and EDX analysis. The information gained about the material properties of the composites, as well as the location of the TS-1 crystals within them, helped in the interpretation of results on diffusion.

### PFG NMR experiments

Fig. 4 and 5 show the diffusion attenuation curves obtained for the two composites, SAC and TS-1, with diffusion times of 180 ms. All attenuation curves revealed non-monoexponential shapes, suggesting the presence of more than one molecular fraction possessing different self-diffusion coefficients during the diffusion time, which is consistent with our previous study.^34^ The diffusion coefficients, calculated based on the attenuation curves of each material, are listed in Table 2.

**Table 2:** Self-diffusion coefficients (*D*_*i*_) and calculated root mean squared displacements (RMSDs) from the fitting of attenuation curves plotted on Fig. 4 and 5 using eqn (1)^a^

Sample	*D*_1_/10^−10^ (m^2^ s^−1^)	RMSD_1_/μm	*D*_2_/10^−11^ (m^2^ s^−1^)	RMSD_2_/μm	*D*_3_/10^−12^ (m^2^ s^−1^)	RMSD_3_/μm
TS-1	2.3 ± 0.4	15.7 ± 0.2	6.5 ± 0.5	8.4 ± 0.4	n.d.	n.d.
SAC	2.1 ± 0.6	12.7 ± 0.3	n.d.	n.d.	2.9 ± 0.5	1.8 ± 0.2
Composite_26	4.0 ± 1.0	19.7 ± 0.4	9.1 ± 0.9	9.9 ± 0.3	2.6 ± 0.5	1.7 ± 0.3
Composite_56	4.0 ± 1.0	19.7 ± 0.4	1.4 ± 0.2	3.9 ± 0.2	3.7 ± 0.6	1.9 ± 0.2

a Determined by PFG NMR.

**Fig. 6:**
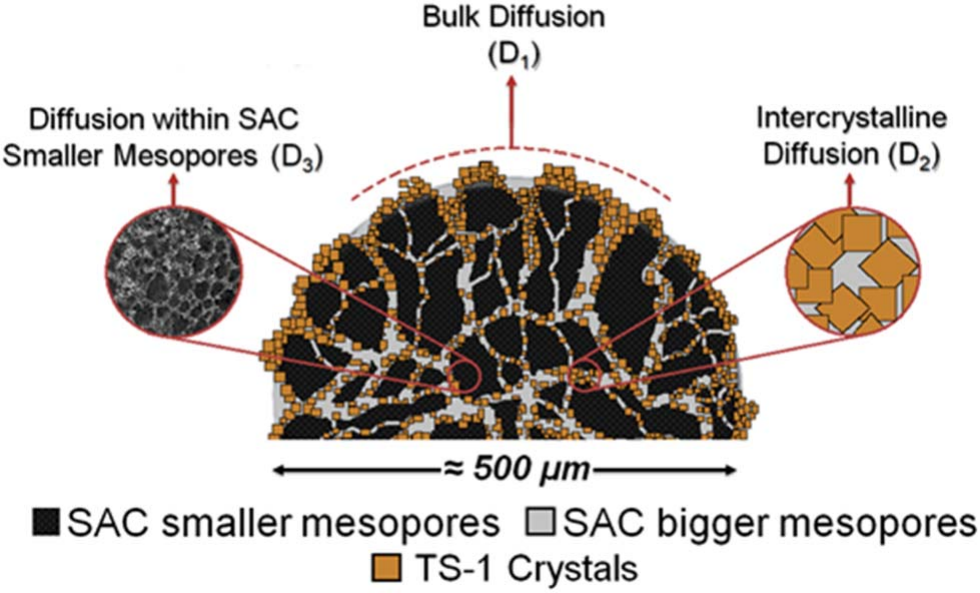
Illustration of suggested regimes of MO diffusion in the presence of a composite particle containing TS-1 and SAC.

Three different diffusion coefficients were calculated based on the attenuation curves. In fact, more can exist, which however can hardly be interpreted without additional information containing spectroscopically resolved signals. These were assigned to the three diffusion regimes illustrated in Fig. 6. The diffusion coefficient representing the slowest observed diffusion regime is denoted as *D*_3_ and is due to MO diffusing within the SAC pores (mainly below 20 nm, see Fig. 2). *D*_3_ (2.9 × 10^−12^ m^2^ s^−1^) is ∼50 times smaller than the diffusion coefficient of MO in the bulk-like phase (*D*_1_ = 2.3 × 10^−10^ m^2^ s^−1^). In this context, it is noteworthy that the diffusion coefficient *D*_3_ determined for pure SAC (2.9 × 10^−12^ m^2^ s^−1^) is comparable to that found in composite materials (2.6 × 10^−12^ m^2^ s^−1^ and 3.7 × 10^−12^ m^2^ s^−1^). The diffusion within the micropores of TS-1 is not detectable because the MO molecule (kinetic diameter ∼2.5 nm (ref. 34)) can hardly enter the micropores of TS-1 (ref. 42) with 0.55 nm pore width.

In addition, relative diffusion coefficients (*D*_rel_ = *D*_*i*_/*D*_bulk_) determined by PFG-NMR can provide information on the interaction between test molecules and catalysts.^43–45^ A decrease in *D*_rel_ for hydrophobic molecules can indicate an increase in the hydrophobicity of the catalyst. Table 1S† shows the calculated *D*_rel_ values for the diffusion coefficients associated with MO diffusion between TS-1 crystals. According to Table 1S,† no significant difference in *D*_rel_ values is observed between Composite_26 and TS-1 (2.28 × 10^−1^*vs.* 2.83 × 10^−1^, respectively). In contrast, the *D*_rel_ of Composite_56 (3.50 × 10^−2^) is approximately eight times lower than that of TS-1 This may be understood as a stronger interaction between the MO and the surface of the TS-1 crystals stacked inside Composite_56. This can be understood as a higher hydrophobicity on the surface of the TS-1 crystals in Composite_56 compared to Composite_26 and TS-1.

However, the effect of slower diffusion in the pore space regime associated to *D*_2_ (intracrystalline diffusion) is more likely to be attributed to a higher degree of filling of the SAC pore space with TS-1 crystals (due to higher TS-1 content). The latter interpretation is also more in line with the observations made concerning population of MO molecules made in the following paragraph. In addition to that a higher hydrophobicity of the composite with lower SAC content (Composite_56) is also counterintuitive.

**Fig. 7:**
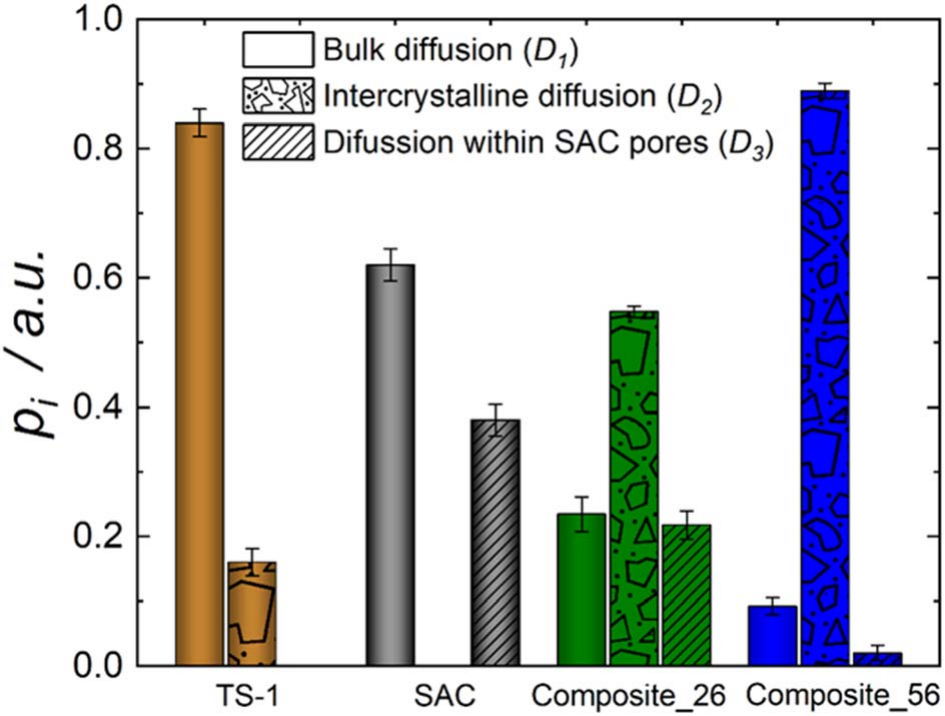
Pre-exponential factor *p*_*i*_ component of eqn (1) in the diffusion of MO for TS-1, SAC, Composite_26, and Composite_56.

Fig. 7 displays the diffusion regimes present in each material and the value of the pre-exponential factor *p*_*i*_ component in eqn (1). In the case of equal transverse relaxation times, this value is proportional to the number of MO molecules diffusing within the corresponding self-diffusion coefficient. In view of the lack of relaxation studies, the interaction of MO with a material usually leads to faster relaxation, leading to an underestimation of the real populations of adsorbed molecules. In addition, the presence of the remaining bulk or bulk-like phases (*e.g.*, as a liquid film on the surface of a crystal) after sample preparation means that without measuring the quantity, the *p*_*i*_-values for different catalysts should not be compared with each other. Thus, the only plausible comparison can be done for the ratios of *p*_2_ and *p*_3_ obtained for Composite_26 and Composite_56.

For the latter composite, this ratio is significantly higher, suggesting that the amount of MO in the intercrystalline space of the TS-1 crystals in the Composite_56 (*p*_2_/*p*_3_ = 43.9) is notably higher than that inside the SAC pores, as compared to the Composite_26 (*p*_2_/*p*_3_ = 2.5). This supports the results of the catalytic experiments that the amount of MO in the pores of SAC (where no active sites are located) decreases notably with increasing TS-1 content. This is likely caused by the TS-1 crystals filling the porous system of the SAC, as also observed in the SEM images (Fig. 3), nitrogen sorption isotherms (Fig. 1), and MIP curves (Fig. 2).

### Catalytic epoxidation of methyl oleate

**Fig. 8:**
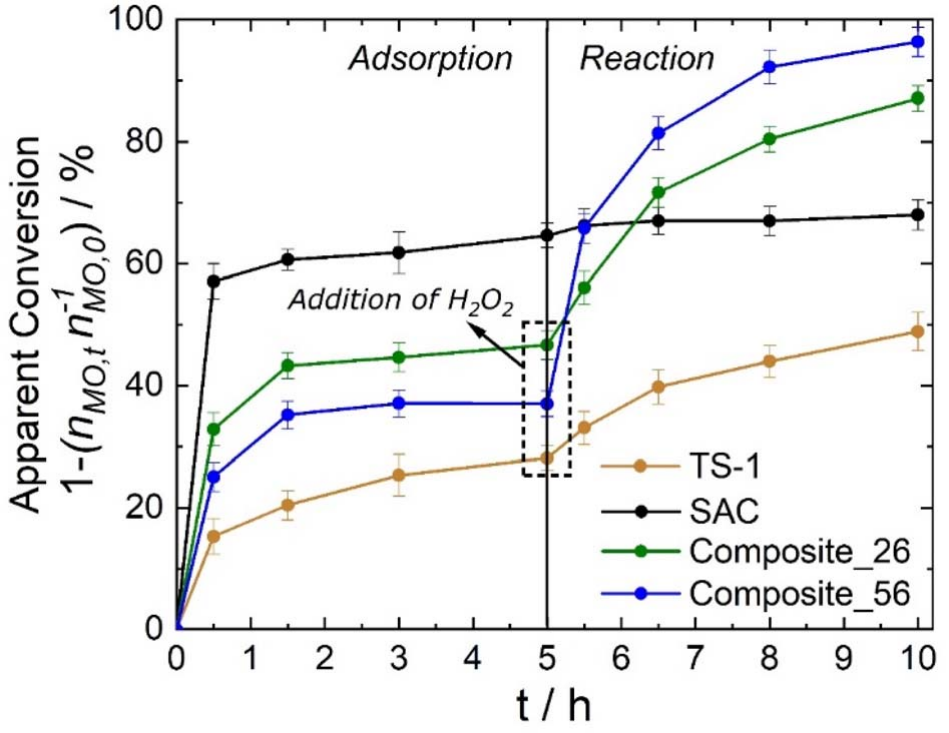
Relative molar MO amount expressed as 1 − (*n*_MO,*t*_*n*_MO,o_^−1^) in the MO epoxidation. (*V*_acetonitrile_ = 10 cm^3^, *C*_MO_ = 0.03 mol L^−1^, *n*_H_2_O_2__/*n*_MO_ = 4 mol mol^−1^, *m*_TS-1_ = 150 mg, *m*_SAC_ = 150 mg, *m*_Composite_ = 150 mg, *T* = 323 K).

The epoxidation of methyl oleate with H_2_O_2_ was carried out to determine the catalytic activity of the synthesized composite materials. Fig. 8 displays the decrease in the MO concentration in the liquid phase expressed as the relative molar MO amount (1 − (*n*_MO,*t*_*n*_MO,o_^−1^)) over the reaction time (*t*). This term considers that the decrease in MO concentration in the liquid phase occurs due to two phenomena: first, the adsorption of MO on the catalyst surface and second, the conversion of MO to eMO as a product of epoxidation. To distinguish between these two phenomena, experiments were carried out in the first 5 h with the catalyst only exposed to MO in the absence of H_2_O_2_ to quantify the adsorption of MO on the catalyst surface. SAC adsorbed 68% of the MO molecules after 5 h, while Composite_26 adsorbed 46%. When the TS-1 weight fraction in the composite was increased (from 26 to 56 wt%), a decrease in MO adsorption was observed (from 46% (Composite_26) to 37% (Composite_56)) which confirms that MO adsorption correlates with SAC content in the composites and thus available specific surface area, as expected. TS-1 also adsorbed a significant number of MO molecules without reaction, decreasing the MO concentration in the liquid phase by approximately 20%.

After 5 h, H_2_O_2_ was added to start the reaction. Composite_56 showed the largest change in the relative molar amount of MO in the first 30 min of the reaction and the highest conversion of MO after 5 h of reaction. Since adsorption equilibrium is reached in the first 5 h, the decrease in the MO concentration in the liquid phase after addition of H_2_O_2_ (*t* > 5 h) is assumed to be caused solely by the catalytic conversion of MO to eMO. These findings already confirm the highest catalytic activity for Composite_56, but also illustrate that when using highly porous catalysts, the adsorption of reactant molecules needs to be considered when determining catalytic activity *via* the reduction of the reactant concentration in the liquid phase.

**Fig. 9:**
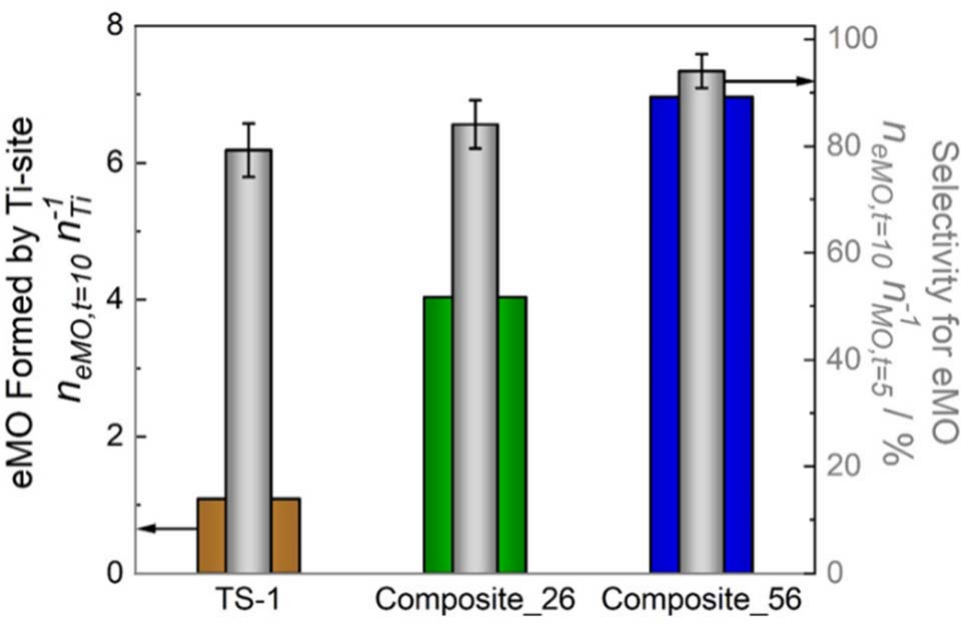
Molar amount of eMO formed per Ti-site after 5 h of reaction expressed as (*n*_eMO,*t*=10_*n*_Ti_^−1^) and relative molar amount of eMO formed expressed as *n*_eMO,*t*=10_*n*_MO,*t*=5_^−1^ after 10 h of reaction over TS-1, and TS-1/SAC composites. (*V*_acetonitrile_ = 10 cm^3^, *C*_MO_ = 0.03 mol L^−1^, *n*_H_2_O2_/*n*_MO_ = 4 mol mol^−1^, *m*_TS-1_ = 150 mg, *m*_Composites_ = 150 mg, *T* = 323 K).

To more accurately quantify the catalytic activity of the three catalysts, the molar amount of eMO formed per Ti-site was calculated from the Ti-content measured by ICP-OES (*n*_eMO,*t*=10_*n*_Ti_^−1^) and the selectivity to eMO in terms of eMO molecules formed per MO converted after the addition of H_2_O_2_ (*n*_eMO,*t*=10_ is *n*_MO,*t*=5_^−1^) are shown in Fig. 9. Here, Composite_56 showed the highest molar amount of eMO produced per Ti-site (6.9 mol mol^−1^) compared to Composite_26 (4.0 mol mol^−1^) and TS-1 (1.1 mol mol^−1^) confirming a more efficient use of the Ti sites.

To exclude drastic differences in the chemical nature of Ti sites, UV-Vis spectroscopy was carried out. The results showed that both composites and TS-1 contained similar Ti species (Fig. 7S†). It is thus assumed that the difference in Ti-site-normalized eMO formation is caused by the different local environment (pore space) in which the TS-1 crystals are situated, as well as a reduction of unwanted diffusion of the MO molecules in the smaller mesopores of the SAC containing no active sites when comparing Composite_26 and Composite_56. Based on these results, it can be concluded that the intercrystalline space between TS-1 crystals is the most reactive environment since the number of MO molecules in this environment compared to the SAC pores (Fig. 7) is directly correlated with higher catalytic activity. Catalytic activity is (as opposed to adsorption, which correlates mainly with specific surface area) linked with a prolonged residence time of the MO in the intracrystalline domain (associated with *D*_2_). This is evidenced by the pre-exponential factor *p*_*i*_ from the PFG NMR-experiments, and can be understood by a partly blocking of the SAC pore space (with no active sites) thus reducing the residence time in the smaller mesopores of SAC (associated with *D*_3_) or, to a less likely degree, as an increase in hydrophobicity and interaction in the intracrystalline space of Composite_56.

This is an important design principle that should be considered when tailoring efficient TS-1 based epoxidation catalysts. This finding could further be explained by the cooperative effect of multiple active sites in close vicinity, which is facilitated by TS-1 stacking, or a positive effect of longer residence time of MO molecules close to the catalytically active TS-1 crystals.

When looking at the selectivity towards eMO formation, Composite_26 and TS-1 exhibited relatively similar values (79 *vs.* 84% respectively), while Composite_56 showed a higher selectivity for eMO (94%). Because only a small amount of unidentified side products was detected in the GC that could not be correlated to more than 3% of MO molecules converted, the discrepancy in the selectivity is attributed to unreactive adsorption, which reduces the MO concentration without the formation of eMO or the adsorption of formed eMO molecules. The slightly higher selectivity of Composite_56 can thus be rationalized by the smaller extent of MO diffusion (and adsorption) inside the SAC pore network.

## Conclusions

TS-1 and composite catalysts containing TS-1 as active phase and spherical activated carbon (SAC) were successfully synthesized with different weight fractions of TS-1 *via* hydrothermal synthesis and employed in the epoxidation of methyl oleate (MO) to epoxy methyl oleate (eMO) using H_2_O_2_. It was shown that the TS-1 crystallites are formed on the external surface of the SAC as well as within its pore system. The SAC is assumed to function similar to a conventional catalyst support for the active site containing TS-1. It thus engineers the arrangement of active sites and space between TS-1 crystals.

The latter was identified to be crucial for the enhanced activity of chemically similar Ti-sites in the composite materials. As evidenced by PFG NMR investigations the higher probability of reactant to be found in the intercrystalline space between TS-1 can be directly correlated with increased Ti-site normalized catalytic activity (up to seven times higher than for parent TS-1, Composite_56). The assumed sportive effect of the SAC was found to be potentially even detrimental to catalytic activity since a higher amount of unreactive sorption and a longer residence time in the mesopore system of the SAC not contributing to reaction leads to lower Ti-site normalized activity (Composite_26). This design principle of combing TS-1 with another material functioning as catalyst support to^34^ engineer the spatial arrangement of the TS-1 crystals, and with that their activity could be an important step towards more active TS-1-based catalysts. It was furthermore pointed out that, especially when combing TS-1 with a high surface area material like SAC, the catalytic activity cannot simply be monitored by analyzing the concentration of reactants and products in the liquid phase since the concentration of these molecules can be veiled by sorption effects.

By PFG NMR, three types of MO diffusion in TS-1/SAC composites were identified and attributed to different spatial diffusion regimes in agreement with literature data. The presented diffusion data demonstrate the feasibility of the application of the PFG NMR technique for the direct determination of diffusion coefficients of high molecular-weight long-chain hydrocarbons and oxygen-functionalized derivatives, such as MO, in composite catalysts. Advanced characterization techniques like PFG NMR also enable a correlation between the statistical location of reactant molecules inside the different pore spaces domains of a composite catalyst under technical relevant conditions and catalytic activity. This knowledge was used to better fundamentally understand the role of the different components in the composite catalyst investigated. It is assumed that similar to a conventional supported metal catalyst, the SAC works as support preventing agglomeration of the supported TS-1 (similar to supported metal particles) and helps to design TS-1 dispersion and the pore space between TS-1 crystals. Especially the latter have been identified to be crucial for catalytic activity.

It should however be noted that, an additional influence of a change in hydrophobicity of the investigated components cannot be fully excluded and is already under investigation in a follow-up study.

## Data availability

The data supporting this article have been included as part of the ESI.†

## Conflicts of interest

There are no conflicts to declare.

## Supplementary Material

RA-015-D4RA08189G-s001
