# Supplementary material for: TS-1/spherical activated carbon composites in the epoxidation of methyl oleate
Source: RSC Adv. 2025 Mar 5;15(9):7111–20. doi: 10.1039/d4ra08189g (PMC11881798; doi:10.1039/d4ra08189g)
Supplement: RA-015-D4RA08189G-s001 [file RA-015-D4RA08189G-s001.pdf]

Supplementary Information (SI) for RSC Advances.
This journal is © The Royal Society of Chemistry 2025

Supplementary material

The Role of Spherical Activated Carbons/TS-1 Composites in the Epoxidation of Methyl Oleate

Adrián Osorio Hernández, Michael Goepel, David Poppitz, Muslim Dvoyashkin, Roger Gläser*

Institute of Chemical Technology, Universität Leipzig, Linnéstr. 3, 04103 Leipzig, Germany.

Corresponding Author

Email: michael.goepel@uni-leipzig.de

Phone: +49 341 97 36333

Fax: +49 341 97 36349

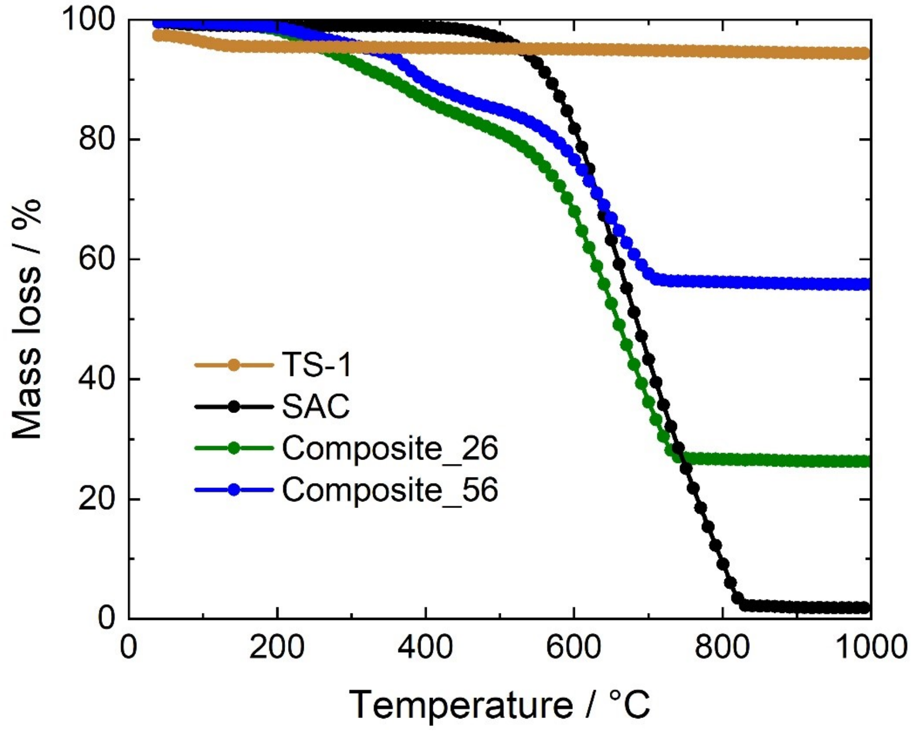

Figure 1S Thermogravimetric analysis (TGA) data showing mass loss of TS-1, SAC, Composite_26 and Composite_56 as a function of temperature between 40 °C and 1000 °C.

Equation 1S: Comparison between the specific pore volume (V_{total}) for Composite_26 and Composite_56 calculated by linear combination ($Value_{LC}$) of the specific pore volumes obtained from TS-1 and SAC and the specific pore volume obtained by nitrogen sorption ($Value_{N2}$) of Composite_26 and Composite_56.

$$Value_{LC}\,Composite\_26 = \left(V_{Total(TS-1)} * Content_{TS-1}\right) + \left(V_{Total\,(SAC)} * Content_{SAC}\right)$$
$$Value_{LC}\,Composite\_26 = \left(0.16\,cm^3g^{-1} * 0.26\right) + \left(1.80\,cm^3g^{-1} * 0.74\right)$$
$$Value_{LC}\,Composite\_26 = \left(0.04\,cm^3g^{-1}\right) + \left(1.33\,cm^3g^{-1}\right)$$
$$Value_{LC}\,Composite\_26 = 1.37\,cm^3g^{-1} \qquad Value_{N2}\,Composite\_26 = 1.02\,cm^3g^{-1}$$

$$Value_{LC}\,Composite\_56 = \left(V_{Total(TS-1)} * Content_{TS-1}\right) + \left(V_{Total\,(SAC)} * Content_{SAC}\right)$$
$$Value_{LC}\,Composite\_56 = \left(0.16\,cm^3g^{-1} * 0.56\right) + \left(1.80\,cm^3g^{-1} * 0.44\right)$$
$$Value_{LC}\,Composite\_56 = \left(0.09\,cm^3g^{-1}\right) + \left(0.79\,cm^3g^{-1}\right)$$
$$Value_{LC}\,Composite\_56 = 0.88\,cm^3g^{-1} \qquad Value_{N2}\,Composite\_56 = 0.53\,cm^3g^{-1}$$

Table 1S: Self-diffusion coefficients (D_i) and relative diffusion coefficients (D_i/D_{bulk}) calculated from the fitting of attenuation curves plotted on Figures 4 and 5.

Sample	D_{bulk} / 10^{-10} ($m^2\,s^{-1}$)	D_2 / 10^{-11} ($m^2\,s^{-1}$)	D_{2_rel}/ 10^{-1} a.u.
TS-1	2.3±0.4	6.5±0.5	2.83
SAC	2.1±0.6	n.d.	---
Composite_26	4.0±1.0	9.1±0.9	2.28
Composite_56	4.0±1.0	1.4±0.2	0.35

[a] determined by PFG NMR

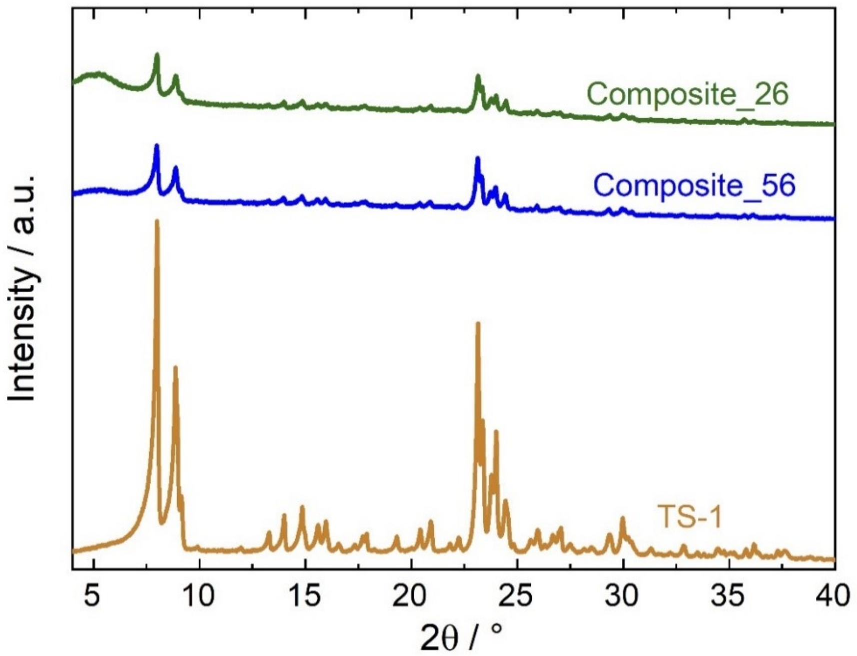

Figure 2S Powder XRD patterns of TS-1, Composite_26, and Composite_56.

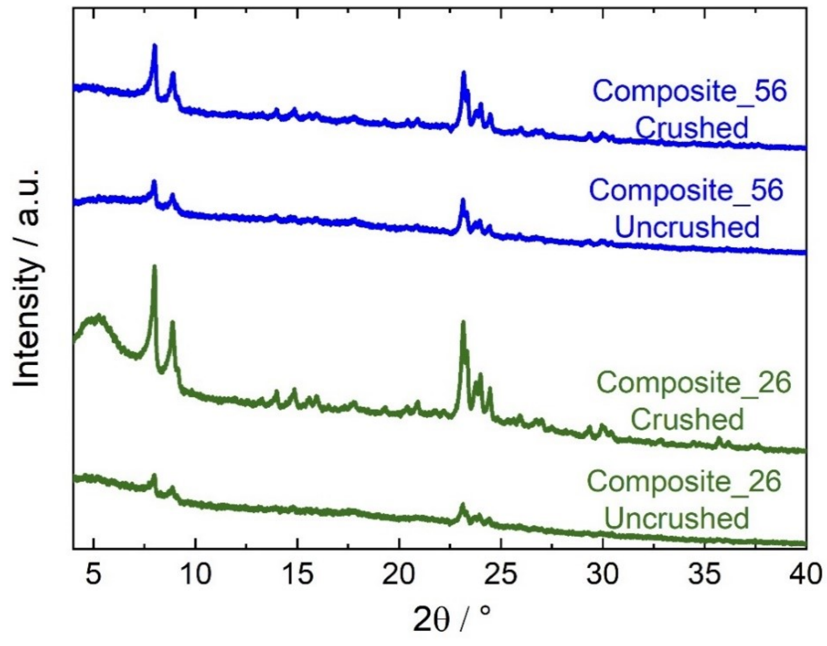

Figure 3S Powder XRD patterns of Composite_26, and Composite_56. (crushed und uncrushed).

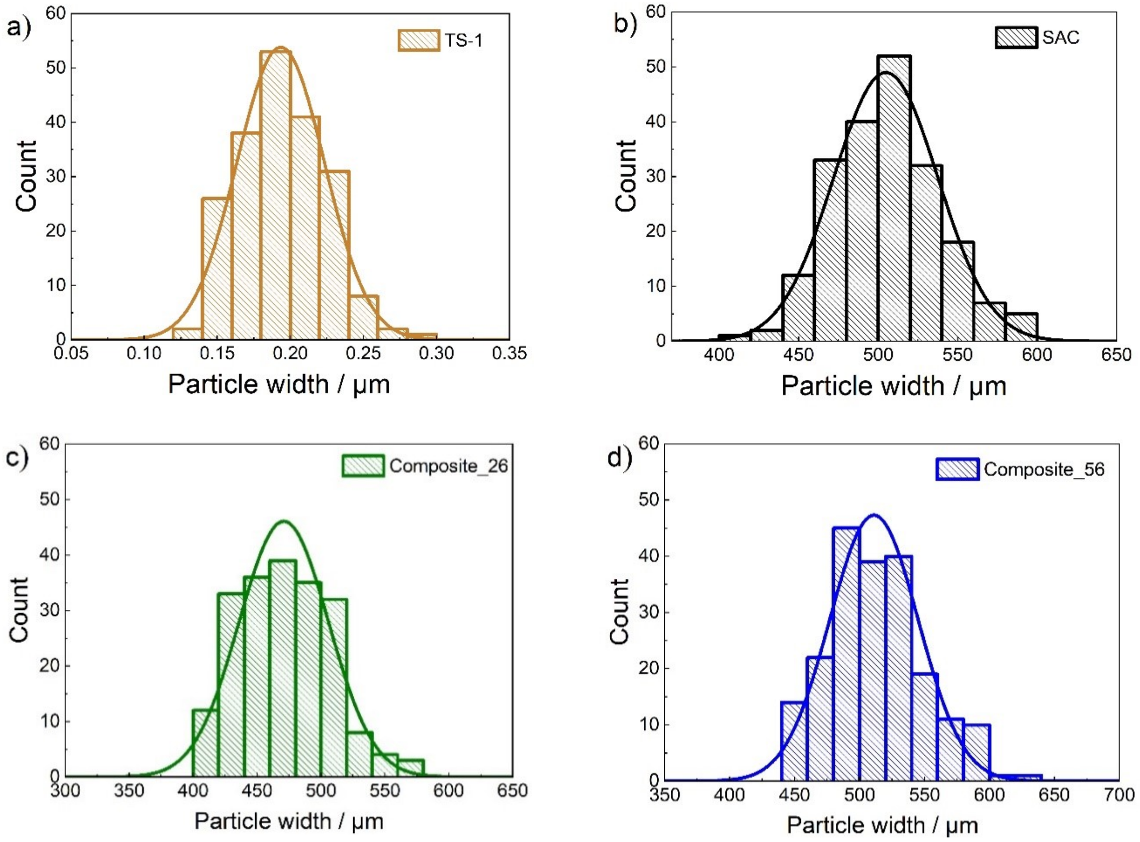

Figure 4S Particle width distribution of TS-1 a), SAC b), Composite_26 c) and Composite_56 d) calculated by taking the average of 100 measurements from the SEM images for each sample.

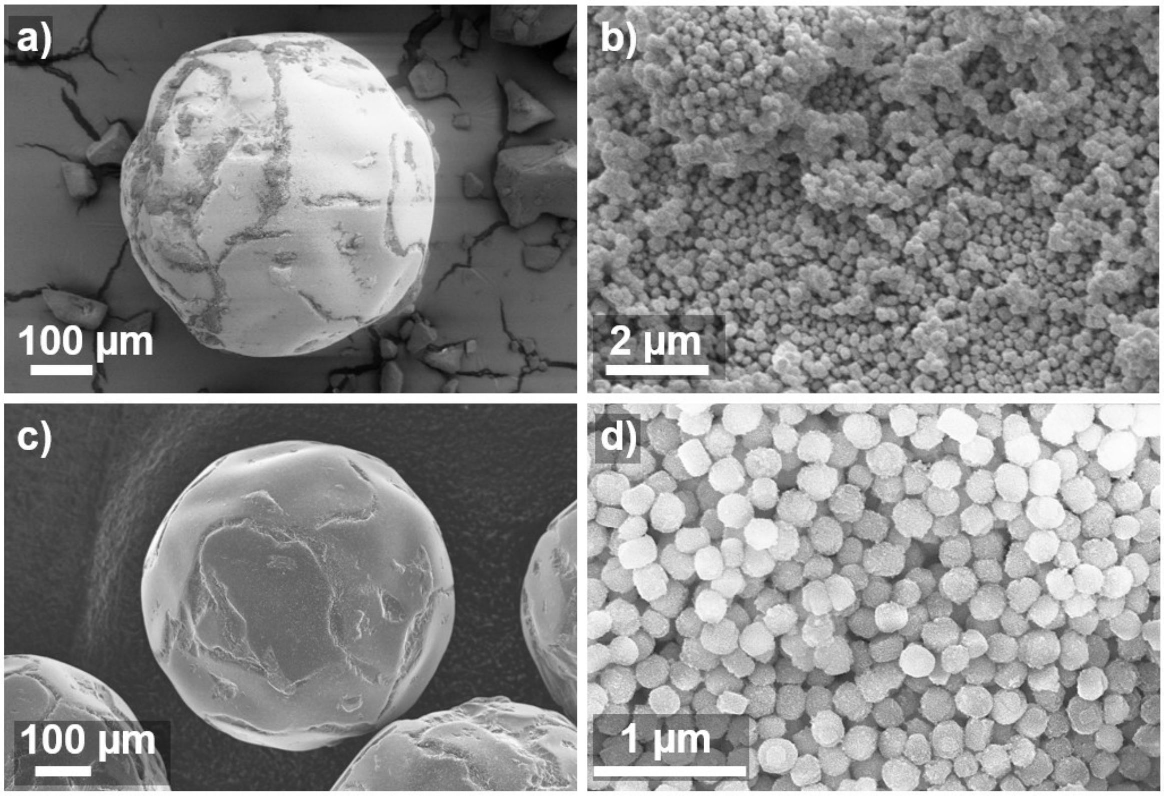

Figure 5S SEM images showing the exterior of a single TS-1/SAC Composite_56 particle (a) and a

close-up view of TS-1 crystallites occupying the surface (b). SAC particle (c) and TS-1 crystals (d).

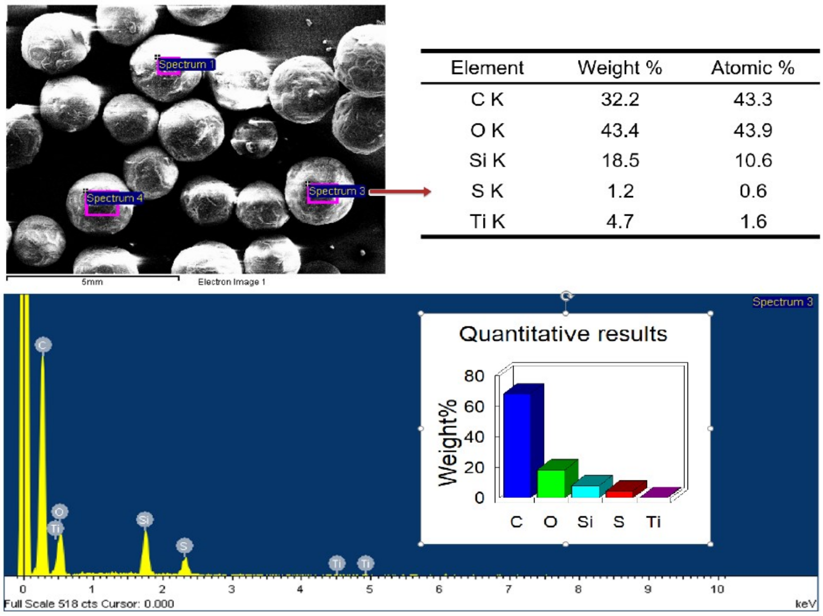

Element	Weight %	Atomic %
C K	32.2	43.3
O K	43.4	43.9
Si K	18.5	10.6
S K	1.2	0.6
Ti K	4.7	1.6

Figure 6S EDX-SEM of a Composite_26 particle.

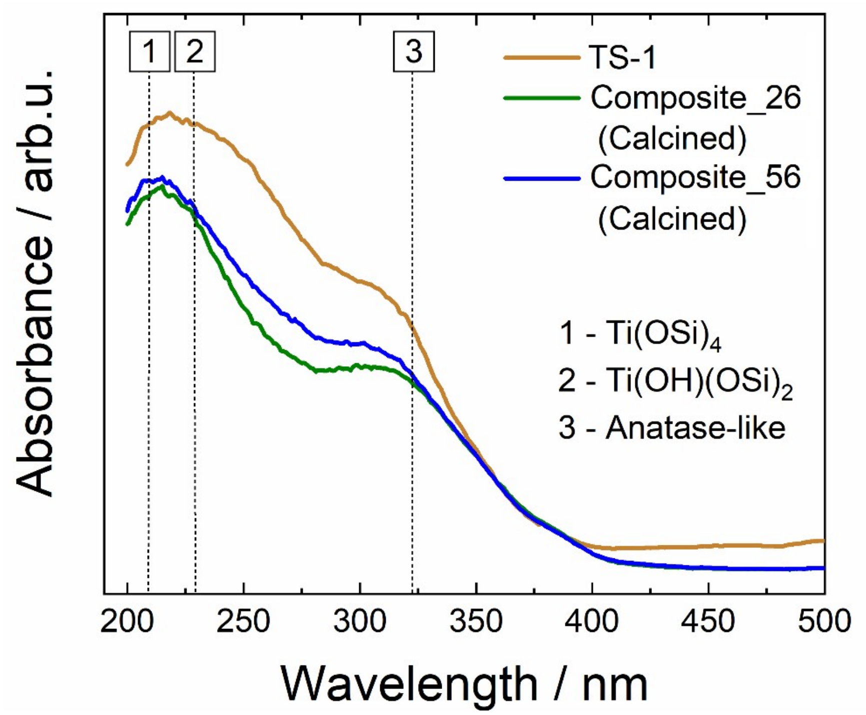

Figure 7S UV-Vis spectra of TS-1, Composite_26 and Composite 56 (Calcined).
